# Epithelial to mesenchymal transition in the liver field: the double face of Everolimus in vitro

**DOI:** 10.1186/s12876-015-0347-6

**Published:** 2015-09-14

**Authors:** Valentina Masola, Amedeo Carraro, Gianluigi Zaza, Gloria Bellin, Umberto Montin, Paola Violi, Antonio Lupo, Umberto Tedeschi

**Affiliations:** 1 Deparment of Medicine, Renal Unit, University Hospital of Verona, 37126 Verona, Italy; 2 Department of General Surgery and Odontoiatrics, Liver Transplant Unit, University Hospital of Verona, 37126, Verona, Italy

## Abstract

**Background:**

Everolimus (EVE), a mammalian target of rapamycin inhibitor, has been proposed as liver transplant immunosuppressive drug, gaining wide interest also for the treatment of cancer. Although an appropriate tolerance, it may induce several adverse effects, such as fibro-interstitial pneumonitis due to the acquisition of activated myofibroblasts. The exact molecular mechanism associated with epithelial to mesenchymal transition (EMT) may be crucial also in the liver context. This work examines the role and the molecular mediators of EMT in hepatic stellate cell (HSC) and human liver cancer cells (HepG2) and the potential role of EVE to maintain the epithelial phenotype rather than to act as a potential initiators of EMT.

**Methods:**

Real time-PCR and western blot have been used to assess the capability of EVE at low-therapeutic (10 nM) and high (100 nM) dose to induce an *in vitro* EMT in HSC and HepG2.

**Results:**

Biomolecular experiments demonstrated that low concentration of EVE (10 nM) did not modify the gene expression of alpha-smooth muscle actin (α-SMA), Vimentin (VIM), Fibronectin (FN) in both HSC and HepG2 cells, whereas EVE at 100 nM induced a significant over-expression of all the three above-mentioned genes and an increment of α-SMA and FN protein levels. Additionally, 100 nM of EVE induced a significant phosphorylation of AKT and an up-regulation of TGF-β expression in HSC and HepG2 cells.

**Discussion:**

Our data, although obtained in an *in vitro* model, revealed, for the first time, that high concentration of EVE may induce EMT in liver cells confirming previous published evidences obtained in renal cells. Additionally, they suggested that mTOR-I should be administered at the lowest dose able to maximize their important and specific therapeutic properties minimizing or avoiding fibrosis-related adverse effects.

**Conclusions:**

In summary, if confirmed by additional studies, our results could be useful for researchers to standardize new therapeutic immunosuppressive and anticancer drugs protocols.

## Background

Standard immunosuppressive strategies after liver transplantation are based on steroids in combination with mycophenolic acid or calcineurin inhibitors (CNI); however their use might be associate with relevant side effects, most of all chronic renal failure, with an incidence of up to 20 % [1]. The mTOR inhibitor and immunosuppressant Everolimus (EVE), in contrast with CNI, is associated with a low nephrotoxicity [2]. EVE belongs to the rapamycin class of drugs that are allosteric inhibitors of mTORC1, an inhibitor of proliferative signal. The main mechanism of action of this drug is the inhibition of mTORC1 complex, a regulatory protein kinase involved in lymphocyte proliferation and other developmental processes [3, 4]. EVE has gained wide interest also in other fields, for example, for the treatment of cancer, switching to less invasive phenotype of tumoral cells and inhibiting angiogenesis [5, 6]. Then, due to this activity it has been proposed in de novo and maintenance liver transplant immunosuppressive protocols to prevent or treat hepatocarcinoma (HCC) recurrence, with survival benefits [7, 8]. From the cellular point of view, interestingly, mTOR signaling is also involved in the mechanism of quiescent hepatic stellate cells (HSC) activation [9]. For this reason, the potential role of mTOR-I in attenuating fibrogenic pathways has been already examined in experimental models, showing a reduced accumulation of extracellular matrix (ECM)-producing cells and ECM components [10]. Then, in consideration of the role played by mTOR-I against proliferation and fibrogenesis, we can suppose that it could alleviate liver fibrosis also in the transplanted graft. Liver fibrosis, which is evident in 75 % of biopsies performed in long-term liver transplant (LTx) survivors, may be promoted by the recurrence of native disease (HCV), hepatotoxicity, de novo disease, non-alcoholic steatohepatitis, chronic rejection and vascular or biliary complications [11]. However, despite evident clinical and experimental advantages of this drug category, mTOR-I may induce the development of several systemic side effects including hematological disorders (anemia, leukopenia and thrombocytopenia), dismetabolism (hyperlipidemia, post-transplant diabetes), lymphedema, stomatitis and fertility/gonadic toxicity [12–14]. Fibrosis related pulmonary adverse effects (e.g., lymphocytic interstitial pneumonitis, bronchiolitis obliterans with organizing pneumonia and focal pulmonary fibrosis) have been also showed in the last years by several reports in oncological and renal transplant patients treated with mTOR-I [15–18]. Our *in vitro* experimental study has already evaluated novel cellular aspects of the potential pro-fibrotic activity of EVE in kidney, showing, for the first time, that epithelial to mesenchymal transition (EMT) in renal tubular cells may be activated by high doses of EVE [19]. It is well known that EMT, a phenotypic conversion of epithelium to a fibroblastic or myofibroblastic phenotype, may have a pivotal role to induce fibrogenesis also in the liver microenviroment [20]. In this context, rearranged ECM by migratory processes induced by EMT appears to be of particular importance also in the mechanism of HCC invasion. In fact, a key event in the development and progression of cancer is the potential of tumor cells to migrate and invade into surrounding tissues. As regards, involvement of PI3K/Akt/mTOR pathway in the interactions between HCC cells and non-parenchymal cells, such as mesenchymal stem cell (MSC), are critical issues for disease progression [21]. Therefore, the aim of our study has been to analyze whether EVE at low-therapeutic [10 nM (corresponding to 3-8 ng/mL serum level)] and high (100 nM) doses might be able to induce in vitro EMT in human hepatoblastoma cells (HepG2) and to activate hepatic stellate cells (HSC). This work prompted us to assess the use of EVE in order to optimize its role in a tailored immunosuppressive regimen.

## Methods

### Cell culture and treatments

HepG2 (human liver cancer cells) were purchased from ATCC and maintained in Dulbecco’s modified Eagle medium (DMEM) (Invitrogen, Carlsbad, CA, USA). The medium was supplemented with 10 % fetal bovine serum (FBS) and 1 % antibiotic penicillin/streptomycin. Cells were maintained at 37 °C, 5 % CO2 humidified incubator.

Hepatic stellate cells (HSC) were isolated from normal male Winstar rats. All animals were purchased from Charles River Laboratories and were housed in the Animal research Facility of Biological-Chemistry Department. They were maintained under a 12-h light–dark cycle and given rat chow and water ad libitum. The animal investigation was in accordance with the National Institute of Health guidelines for the care of Laboratory animals and was approved by the local committee for supervision of animal experiments at the University Hospital of Padova, Italy. This is over and above adherence to general guidelines.

HSCs were isolated from normal livers according to a modified Zhang method [22]; livers were perfused with a calcium-free buffer solution to wash out the blood, subsequently with a wash solution (calcium-free solution with CaCl_2_ 0.294 g/L, MgSO_4_ 0.097 g/L) and finally incubated in a digestion buffer (collagenase 0.6 g/L in Gey’s Balanced Salt Solution with Ca2^+^and Mg2^+^, pH 7.5) for 1 h (37 °C, CO2 5 %) to digest the extracellular matrix. The organs were cut into small pieces and subjected to homogenization to produce a single cell-suspension, which was centrifuged at 1450 × g for 18 min in 12 % (wt/vol) Nycodenz gradient to obtain a pure HSC fraction. Both the number and viability of HSCs were determined using the trypan blue dye exclusion test.

Collected HSCs were washed with Hank’s balanced salt solution and resuspended at a concentration of 1 × 10^5^ cells/ml, in DMEM (supplemented with 20 % FBS and 1× antibiotic solution) and cultured on collagen type I coated well plates (10 μg/ml) in a 5 % CO2-humidified atmosphere. Serum-starved cells were subsequently detached for experiments using trypsin/EDTA solution.

All cell experiments were performed between passages 3 and 4.

Everolimus was kindly provided by Novartis (Basel, Switzerland) and dissolved in DMSO according to the manufacturer’s instructions.

HepG2 and HSC cells were plated in complete growth medium and then starved for 24 h in serum free medium. Cells were cultured for additional 1, 6 or 24 h with 10 or 100 nM EVE and then assayed for gene and protein expression. To evaluate the contribution of AKT-activation cells were pretreated (1 h before) with LY294002 (25 μM).

### Gene expression analysis

Total RNA was extracted from the cell monolayer using the GenElute Mammalian Total RNA Miniprep kit (Sigma-Aldrich) including DNase treatment (DNase70; Sigma). Yield and purity were assessed using Nanodrop (EuroClone) and Agilent 2100 Bioanalyzer, respectively.

Total RNA from each sample was reverse-transcribed into cDNA using SuperScript II reverse transcriptase (Invitrogen). Real-time PCR were performed on an ABI-Prism 7500 using Power SYBR Green Master Mix 2 (Applied Biosystems). A quantitative analysis was performed to evaluate the expression of α-smooth muscle actin (α-SMA), vimentin (VIM), fibronectin (FN) and TGFβ normalized to GAPDH. The comparative Ct method (ΔΔCt) was used to quantify gene expression, and the relative quantification was calculated as 2^-ΔΔCt^. Melting curve analysis was performed to check for any presence of non-specific amplification products. The forward and reverse primer sequences are reported in Table 1 [23].

**Table 1:** Primers sequence list

Human	Forward	Reverse
α-SMA	GAAGAAGAGGACAGCACTG	TCCCATTCCCACCATCAC
FN	GTGTGTTGGGAATGGTCGTG	GACGCTTGTGGAATGTGTCG
VIM	AAAACACCCTGCAATCTTTCAGA	CACTTTGCGTTCAAGGTCAAGAC
TGF-β	CGTGGAGCTGTACCAGAAAT	GATAACCACTCTGGCGAGTC
GAPDH	ACACCCACTCCTCCACCTTT	TCCACCACCCTGTTGCTGTA
Rat	Forward	Reverse
α-SMA	GAAGAAGAGGACAGCACTG	TCCCATTCCCACCATCAC
FN	CCAGGCACTGACTACAAGA	CATGATACCAGCAAGGACTT
VIM	TGACCGCTTCGCCAACTA	CGCAACTCCCTCATCTCCT
TGF-β	ATACGCCTGAGTGGCTGTCT	TGGGACTGATCCCATTGATT
GAPDH	TGAGGACCAGGTTGTCTC	TCCACCACCCTGTTGCTGTA

### Western blot

The cells were treated in lysis buffer (50 mMTris-HCl, pH 5.0, 150 mMNaCl,0.5 % Triton X-100) with Complete protease inhibitor mixture (Roche Applied Science) and phosphatase inhibitor cocktails 1 and 2 (Sigma-Aldrich) and quantified using the Bio-Rad protein assay. Equal amounts of proteins were treated in reducing sample buffer and denatured for 10 min at 100 °C. Protein samples were then resolved in 10 % SDS-PAGE and electro transferred to nitrocellulose membranes. Nonspecific binding was blocked for 2 h at room temperature with nonfat milk (5 %) in TBST buffer (50mMTris-HCl, pH 7.4, 150 mM NaCl, 0.1Tween 20). Membranes were exposed to primary antibody overnight at4 °C and incubated with a secondary peroxidase-conjugated antibody for 1 h at room temperature. The primary antibodies were sc-9068 for FN, sc-130619 for alpha-SMA, sc-25778 for GAPDH, sc-7985-R for pAKT (SantaCruz Biotechnology) and GTX121937 for AKT (GeneTex). The signal was detected with the Super Signals West Pico Chemiluminescent substrate solution (Pierce) according to the manufacturer’s instructions. Bands of three independent experiments were analyzed by the Image J software (National Institutes of Health) to obtain mean values.

## Results

### High doses of EVE activate HSC

**Fig. 1:**
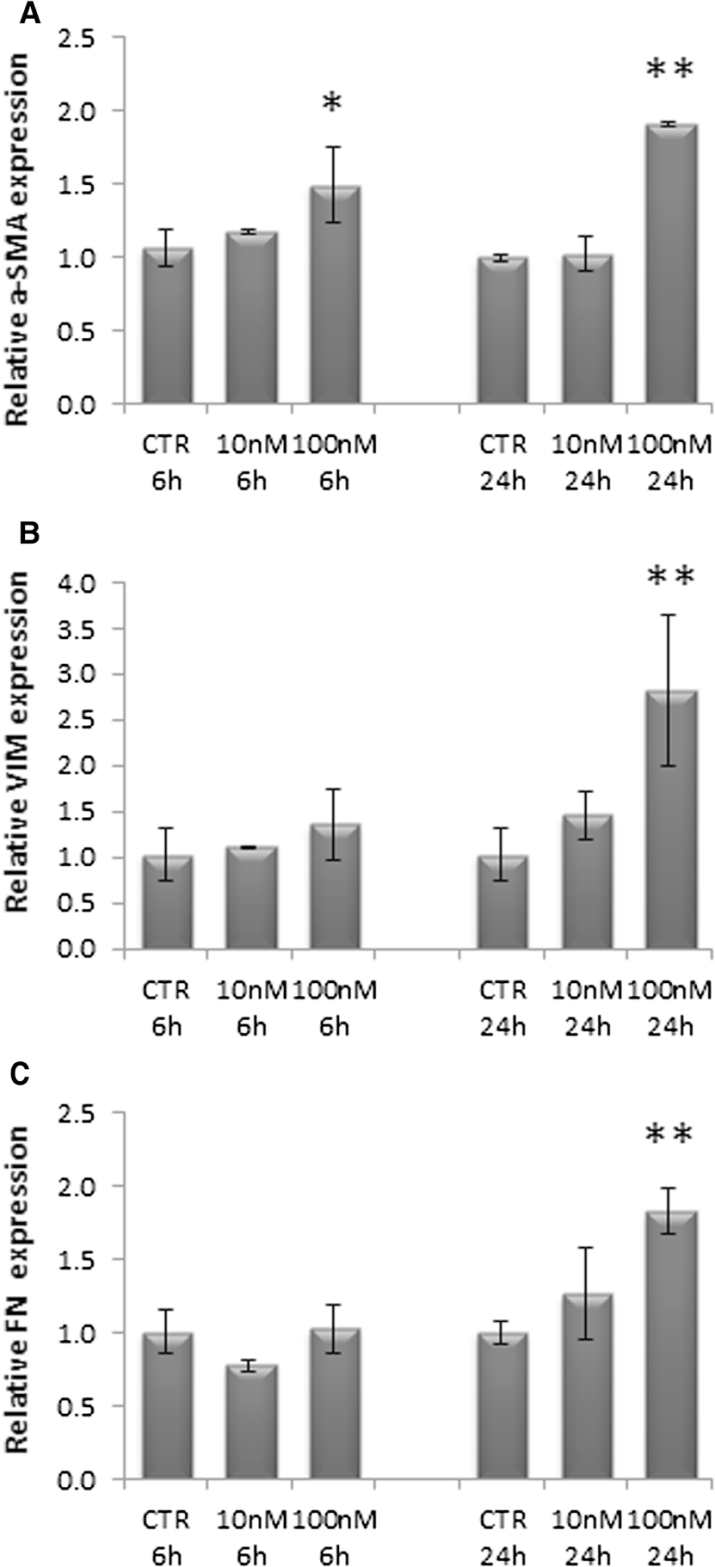
Relative α-SMA, VIM and FN gene expression in HSC treated with EVE. Histograms represent relative gene expression of **a**) α-SMA, **b**) VIM and **c**) FN evaluated by real-time PCR in HSC cells treated or untreated with EVE (10 or 100nM) for 6 or 24 h. Results were normalized to GAPDH expression. Mean ± S.D. of two separate experiments (error bars) performed in triplicate. *p < 0.05; **p < 0.001 vs untreated cells

**Fig. 2:**
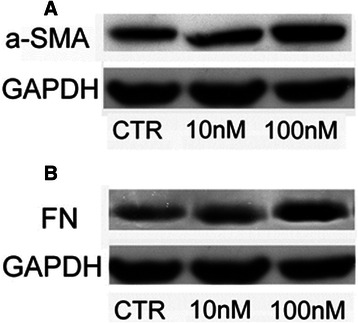
α-SMA and FN protein expression in HSC treated with EVE. Western blot analysis was performed to evaluate **a**) α−SMA and **b**) FN expression in HSC cells treated or untreated with EVE (10 or 100 nM) for 24 h. GAPDH was included as loading control. Representative blot of three independent experiments

To characterize the effect EVE on activation of hepatic stellate cells, we treated primary cultured HSC cells with different doses of EVE. HSC were analyzed at low passages (3 or 4) to limit the spontaneous activation. The treatment with the therapeutic concentration 10nM did not modify the gene expression of α-SMA, VIM and FN both after one, six and 24 h. Differentially, the treatment with 100 nM EVE significantly induced a 1.49 and 1.91 fold increase in α-SMA expression respectively after 6 and 24 h. Moreover the treatment for 24 h with 100 nM EVE induced a 2.82 and 1.83 fold increase respectively in VIM and FN expression (Fig. 1). The treatment with 100 nM EVE induced also a significant increase in α-SMA and FN protein levels (Fig. 2).

### High doses of EVE activate the EMT program in hepatocarcinoma cells

**Fig. 3:**
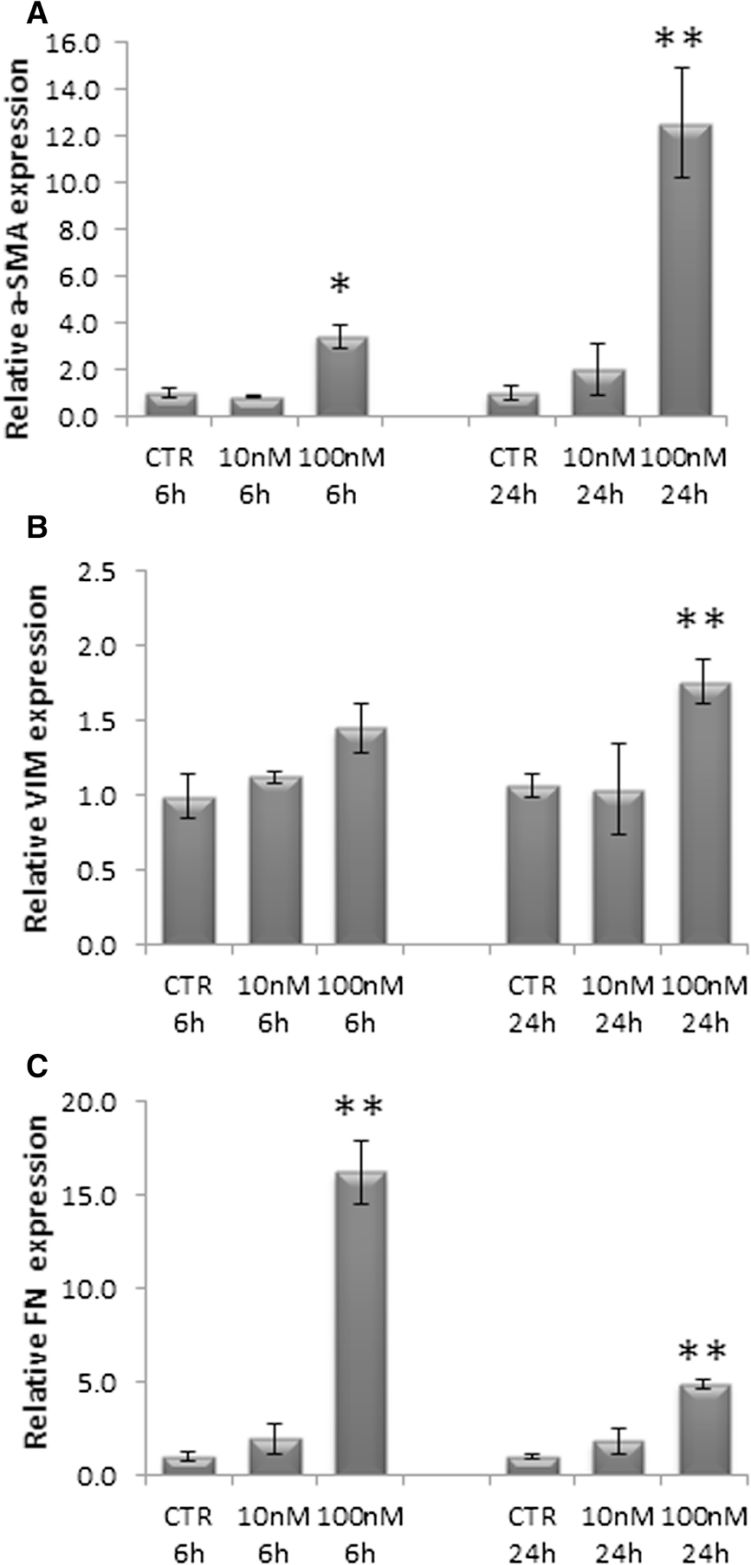
Relative α-SMA, VIM and FN gene expression in HepG2 treated with EVE. Histograms represent relative gene expression of **a**) α-SMA, **b**) VIM and **c**) FN evaluated by real-time PCR in HepG2 cells treated or untreated with EVE (10 or 100 nM) for 6 or 24 h. Results were normalized to GAPDH expression. Mean ± S.D. of two separate experiments (error bars) performed in triplicate. *p < 0.05; **p < 0.001 vs untreated cells

**Fig. 4:**
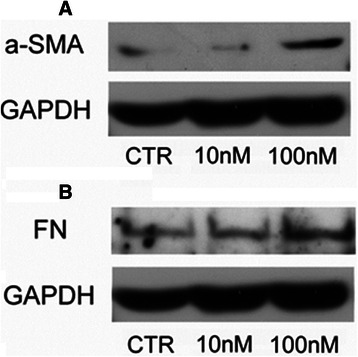
α-SMA and FN protein expression in HepG2 treated with EVE. Western blot analysis was performed to evaluate **a**) α-SMA and **b**) FN expression in HepG2 cells treated or untreated with EVE (10 or 100 nM) for 24 h. GAPDH was included as loading control. Representative blot of three independent experiments

Since also other liver components could be affected by these properties of EVE at high dose, and since the establishment of a fibrotic condition is a prerequisite in liver cancer development we evaluated the effect of different EVE doses in HepG2 cells. The concentration 10 nM Eve did not induce alterations in α-SMA, VIM and FN gene expression. On the other hand 100 nM EVE induced a significant up-regulation of all the three mesenchymal markers already after six hours (Fig. 3). Protein expression analysis confirmed an over-expression of α-SMA and FN induced by 100 nM EVE (Fig. 4).

### TGF-β regulation

**Fig. 5:**
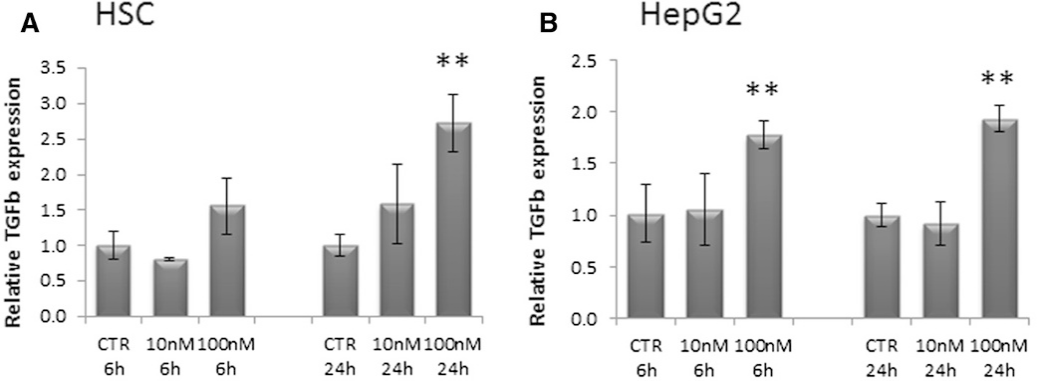
Relative TGF-β gene expression in HSC and HepG2 cells treated with EVE. Histograms represent relative gene expression of TGF-β evaluated by real-time PCR in **a**) HSC and **b**) HepG2 cells treated or untreated with EVE (10 or 100 nM) for 6 or 24 h. Results were normalized to GAPDH expression. Mean ± S.D. of two separate experiments (error bars) performed in triplicate. *p < 0.05; **p < 0.001 vs untreated cells

TGF-β, which is one of the most important factors involved in EMT and fibrosis, has also been showed to be potentially regulated by EVE in a dose-dependent manner. Our results demonstrated that 10 nM EVE did not influence TGF-β expression, while 100 nM EVE up-regulated TGF-β expression already after six hours both in HSC and HepG2 cells (Fig. 5).

### AKT activation induced by EVE

Since several studies suggested that mTORC1 inhibition by EVE may induce AKT activation leading to the attenuation of its therapeutic effects [24] and since AKT is an important signaling pathway involved in EMT we evaluated the effect of EVE in AKT activation.

**Fig. 6:**
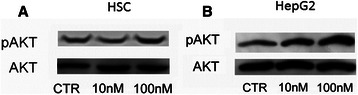
AKT phosphorylation in HSC and HepG2 cells treated with EVE. Western blot analysis was performed to evaluate pAKT and AKT expression in **a**) HSC and **b**) HepG2 cells treated or untreated with EVE (10 or 100 nM) for 1 h. Representative blot of three independent experiments

**Fig. 7:**
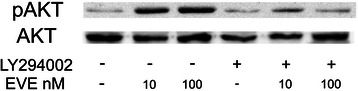
Inhibition of AKT phosphorylation in HepG2 cells. Westernblot analysis was performed to evaluate pAKT and AKT expression inHepG2 cells treated or untreated with EVE (10 or 100 nM) for 1 hand pretreated (1 h before) with 25 μM LY294002

**Fig. 8:**
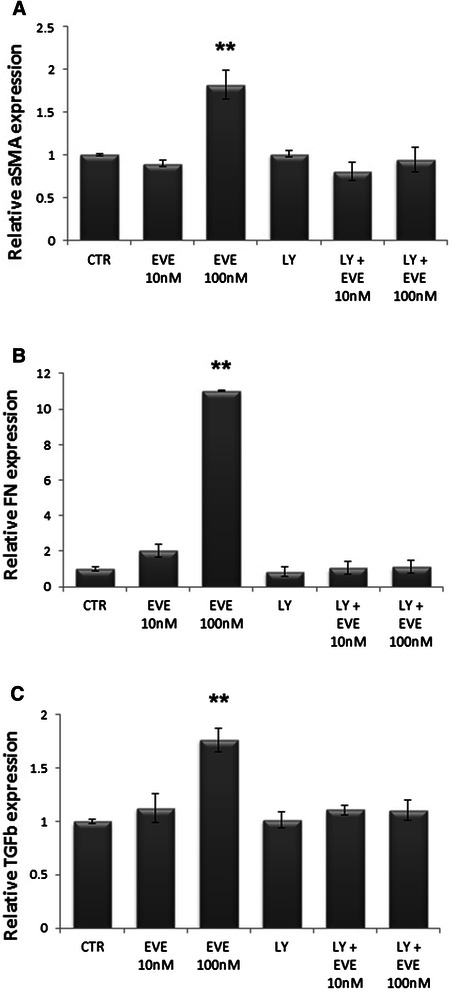
Regulation of α-SMA, FN and TGF-β gene expression in HepG2 treated with EVE by AKT activation. Histograms represent relative gene expression of **a**) α-SMA, **b**) FN and **c**) TGF-β evaluated by real-time PCR in HepG2 cells treated or untreated with EVE (10 or 100nM) for 6 in presence or not of the inhibitor LY294002. Results were normalized to GAPDH expression. Mean ± S.D. of two separate experiments (error bars) performed in triplicate. **p < 0.001 vs untreated cells

Western blot analysis of pAKT/AKT revealed that a high dosage of EVE (100 nM) induced a phosphorylation of AKT both in HSC and HepG2 cells whereas therapeutic concentrations (10 nM) had no effects (Fig. 6). Moreover to prove that the activation of EMT program in HepG2 cells is mediated by the AKT pathway we inhibited its activation whit LY 294002 (Fig. 7). Gene expression analysis confirmed that the up-regulation of α-SMA, FN and TGF-β induced by 100 nM EVE is prevented by the inhibition of AKT (Fig. 8).

## Discussion

Since their introduction in liver transplant therapy, mTOR inhibitors have been considered promising immunosuppressant due to their relatively low nephrotoxicity [2, 25]; the use of EVE is gaining acceptance as maintenance in de novo liver transplant, in cases of renal dysfunction as a CNI-sparing regimen and in patients transplanted for HCC [25, 26]. The main mechanism of action of this drug is the inhibition of cell signaling through the PI3K/Akt/mTOR pathway [27, 28]. The PI3K/AKT/mTOR signaling pathway represents a promising target due to its central role in regulating different cellular activities, including growth, survival, movement, differentiation and metabolism [29]. Since the main mechanism of action of EVE is the inhibition of mTORC1 complex, its efficacy might be due to a feedback activation of upstream PI3K signaling, leading to AKT hyperactivation [30]. AKT feedback is thought to be mediated by the inhibition of p70S6 kinase activity and different mechanisms of its activation have been proposed [31]. An increase in the phosphorylation of receptor tyrosine kinases, i.e. EGFR, HER2, HER3 among others, following treatment with mTOR-I has been demonstrated and may represent an IRS-1-(insulin receptor substrate 1) independent way of increased AKT signaling. As regards, Rosich et al reported that the activity of EVE is limited by AKT re-phosphorylation and suggested that targeting the PI3K/AKT/mTOR pathway at multiple levels is likely to be a more effective strategy for the treatment of mantle cell lymphoma [32]. Thus, despite the known antifibrotic EVE activity, an aberrant signaling with a negative feedback loop might result in the AKT activation following mTOR inhibition; this might be translated into the activation of cellular processes such as EMT. In fact, as we previously showed, high concentrations of EVE could induce renal fibrosis by activating tubular EMT [19]. Therefore, to evaluate whether EVE treatment was able to interfere with the liver extracellular microenvironment by inducing EMT in rat stellate cell (HSC) and human liver cancer cell lines (HepG2), we measured, by RT-PCR, changes in expression level of genes encoding for well known EMT markers (α-SMA, Fibronectin and Vimentin) after both low-therapeutic (10 nM) and high (100 nM) doses; low dose has been considered therapeutic as it corresponds to the usual serum range (3-8 ng/mL) in the liver transplant setting. Our results demonstrated that the treatment with the therapeutic concentration 10 nM did not modify the gene expression of α-SMA, VIM and FN both after six and 24 h. These data confirm that EVE remains a potential inhibitor of liver fibrosis at therapeutic concentration, inhibiting collagen production by activated stellate cells, as well as cell contraction. Therefore, though therapeutic dose might attenuate the activation of primary stellate cells to their activated form, the treatment with 100 nM EVE (which is higher than the recommended through level in the liver transplant) significantly induced an increase in α-SMA, VIM and FN expression. Similarly, as regards HepG2 cell line, the therapeutic dose (10 nM EVE) did not induce alterations in α-SMA, VIM and FN gene expression. On the other hand 100 nM EVE induced a significant up-regulation of all the three mesenchymal markers already after six hours (Fig. 3).

These data suggest that progression of liver fibrosis represents a complex process that involves several pathological events (HSC activation, inflammation etc); on this basis the inhibition of multiple pathological processes using a single drug might be uneffective. Then, though our results are in line with several published papers reporting potential anti-fibrotic liver properties of both mTOR-I [9], however different unknown mechanisms could influence pAKT/AKT signal. Western blot analysis of pAKT/AKT confirmed that a high dosage of EVE (100 nM) induces the phosphorylation of AKT both in HSC and HepG2 cells causing EMT.

Moreover, TGF-β expression is up-regulated by high dose of EVE (100 nM EVE) after six hours both in HSC and HepG2 (Fig. 6). Since EVE concentration (e.g, 100 nM) able to induce EMT is higher compared to that normally used in standard immunosuppressive regimens, our data might not imply any clinical consequence.

Despite this consideration, in our opinion, our results highlight a relevant aspect of this drug. Interestingly, although EMT-related effects were reached in our model only with very high concentration of this drug (beyond the recommended clinical serum level) we cannot exclude that other different stimuli or patients with a genetic predisposition could present this condition after exposure to lower or therapeutic dose of EVE. This assumption is in line with a recent work published by Xu et al describing a pro-fibrotic effect of mTOR inhibitors in lung epithelial cells [33].

Altogether, our data, although obtained by an in vitro model, reveal new biological/cellular aspects of the liver and systemic pro-fibrotic machinery induced by EVE treatment; if confirmed by additional studies, they could be useful for researchers to develop new therapeutic strategies that may prevent/minimize the systemic fibrotic adverse effects induced by high doses EVE therapy. A negative feedback loop resulting in the activation of AKT following mTOR inhibition might also represent a potentially unfavorable event resulting from cancer treatment with mTOR inhibitors. As regards, the phosphorylation of AKT pathway at S473 is detected in up to 71 % of HCC samples, and associated with invasion, metastasis, and vascularization of HCC as showed by Chen e al [34]. On the other hand, the EMT program sustained by AKT feedback-activation may interfere with malignant cell/microenvironment interactions, thanks to its involvement in essential processes such as migration, invasiveness and cytokine signaling. Though laboratory cell experimental studies are good models to study the genetics of cancer, however they might not permit to clarify and to understand the epigenetic surrounding the carcinogenesis. Despite this consideration, that aspect is linked to several clinical trials sustained at present whose emerging data indicate that RAD001, even well tolerated, has only moderate antitumor efficacy in HCC patients [35, 36].

The mechanisms by which immunosuppressive drugs can influence neoplasia is complex; and, as suggested by Bugelski et al, the identification of the relative risk for patients from preclinical data remains problematic. On the other hand, classifying immunosuppressive drugs based on their mechanism of action and hazard identification from preclinical studies to monitor carcinogenic risk may be a feasible way to manage patient safety [37].

In fact, antineoplastic properties of mTOR-I seem to be limited and might be enhanced by the contemporary inhibition of the crosstalk among mTORC1, mTORC2 and Phosphatidylinositol-3 kinase (PI3K)/AKT [8, 38].

Our in vitro study reveals new biological/cellular aspects of the pro-fibrotic activity of EVE and it demonstrates, for the first time, that an EMT program in parenchymal and non-parenchymal liver cells may be activated by high doses of this drug. Additionally, these data, confirming previous evidence [39], suggest that clinicians should administer the adequate dosage of EVE in order to increase efficacy and reduce adverse effects. Further studies are needed about PI3K/Akt/mTOR pathway may prevent the EMT process EVE-dependent and optimized its antitumor effect.

## Conclusions

Liver EMT and consequent fibrosis is characterized by an orchestrated, highly regulated process. Altogether, our data, although obtained in an *in vitro* model, reveal new biological/cellular aspects of the liver and systemic pro-fibrotic machinery induced by high-dose EVE treatment; if confirmed by additional studies, they could be useful for researchers to develop new therapeutic strategies that may increase efficacy and reduce potential adverse affects of EVE also in the field of cancer.

## Availability of data and materials

Not applicable.
